# Perceptions and Experiences of Oral Health Management in Dementia: A Qualitative Systematic Review

**DOI:** 10.1093/geroni/igaf122.3825

**Published:** 2025-12-31

**Authors:** Jia Yin Ruan, Jin Su, Xiang Qi, Weiyu Mao, Yuning Xie, Junwen Yu, Jing Wang, Bei Wu

**Affiliations:** New York University, New York, New York, United States; New York University, New York, New York, United States; New York University, New York, New York, United States; University of Nevada, Reno, Reno, Nevada, United States; Renmin University of China, Beijing, China (People’s Republic); Fudan University, Shanghai, China (People’s Republic); University of New Hampshire, Durham, New Hampshire, United States; NYU Shanghai, Shanghai, China (People’s Republic)

## Abstract

People with dementia often experience oral health problems, and challenges have been identified in managing oral health within this population. Although qualitative studies have explored these challenges from the perspectives of patients, caregivers, and healthcare professionals, there remains a lack of studies that synthesize perceptions and experiences of oral health management from multiple perspectives across diverse contexts. This study aimed to address this gap through a qualitative systematic review. A comprehensive literature search was conducted across eleven databases, covering their inception through July 2025, to identify relevant qualitative studies. Gray literature search was conducted using OpenGrey, Google, and Google Scholar. Hand searches of the reference lists of the included studies were conducted. The Critical Appraisal Skills Programme checklist for qualitative studies was applied to measure the quality of the studies. Data were extracted and analyzed using thematic synthesis. The degree of confidence in the findings was evaluated using the Grading of Recommendations Assessment, Development, and Evaluation Confidence in Evidence approach. A total of 15 studies were included, and three themes were identified: (1) realizing the importance of oral health in dementia as an evolving process, (2) acknowledging the distinct needs of oral health management in dementia, and (3) developing oral health management in dementia across multiple domains. This study reveals stages in dementia oral health management and highlights its unique needs. Future studies should address these unmet oral health needs and explore how caregivers and healthcare professionals can more effectively support oral health management in both clinical practice and research.

